# Mycobacterial glycolipid Di-*O*-acyl trehalose promotes a tolerogenic profile in dendritic cells

**DOI:** 10.1371/journal.pone.0207202

**Published:** 2018-12-10

**Authors:** Alejandro Magallanes-Puebla, Patricia Espinosa-Cueto, Luz M. López-Marín, Raul Mancilla

**Affiliations:** 1 Departamento de Inmunología, Instituto de Investigaciones Biomédicas, Universidad Nacional Autónoma de México, México City, México; 2 Departamento de Ingeniería Molecular de Materiales¸ Centro de Física Aplicada y Tecnología Avanzada, Universidad Nacional Autónoma de México, Querétaro, México; King's College London, UNITED KINGDOM

## Abstract

Due to prolonged coevolution with the human being, *Mycobacterium tuberculosis* has acquired a sophisticated capacity to evade host immunity and persist in a latent state in the infected individual. As part of this evolutive process, mycobacteria have developed a highly complex cell wall that acts as a protective barrier. Herein we studied the effects of Di-*O*-acyl trehalose, a cell-wall glycolipid of virulent mycobacteria on murine bone marrow-derived dendritic cells. We have demonstrated that Di-*O*-Acyl-trehalose promotes a tolerogenic phenotype in bone marrow-derived murine DCs activated with mycobacterial antigens and Toll-like receptor agonists. This phenotype included low expression of antigen presentation and costimulatory molecules and altered cytokine production with downregulation of IL-12 and upregulation of IL-10, an anti-inflammatory cytokine. Additional markers of tolerogenicity were the expression of Indoleamine 2,3-dioxygenase and CD25. Furthermore, Di-*O*-Acyl-Trehalose promoted the expansion of FoxP3^+^ regulatory T lymphocytes. A better understanding of mycobacterial cell-wall components involved in the evasion of immunity is a prerequisite to designing better strategies to fight tuberculosis.

## Introduction

The development of new strategies to fight TB requires a better understanding of the actions that *Mycobacterium tuberculosis* (Mtb) displays to evade host immunity. In the response against mycobacteria, innate and acquired immunity mechanisms participate, and the dendritic cells (DCs) play a central role. DCs in the vicinity of alveoli and tuberculous lesions capture bacilli and migrate to draining lymph nodes where they present antigen to T cells to initiate adaptative T cell response [1, 2]. In *in vitro* studies, DCs exposed to the bacilli develop a well defined immunogenic phenotype with upregulation of molecules involved in T cell activation and production of proinflammatory cytokines [2, 3]. However, in other studies, exposure to mycobacteria or their components impairs their activation and ability to drive T cell proliferation [4, 5]. More recently it has been recognized that DC can participate actively in peripheral tolerance. This ability is related to several factors including phenotype, maturation state, and exposure to inflammatory and microbial factors [6, 7]. It also matters how the DCs sense foreign antigens; activation via Toll-like receptors (TLR), mainly TLR-4 and TLR-2 induce an immunogenic response while lectin-like receptors favor tolerogenic DCs that produce anti-inflammatory factors, and activate regulatory T lymphocytes [8, 9].

Given the prolonged coevolution with humans, the tubercle bacillus has developed the capacity to infect and persist in a dormant state in host tissues. This ability depends at least in part on its highly complex cell envelope. Cell wall components, particularly glycolipids located in the outer part of the cell wall play a leading role in the virulence of Mtb [10, 11, 12]. Some of these glycolipids target antigen presentation cells thus compromising the development of T cell immunity. DCs activated with ManLAM exhibit reduced expression of MHC, CD83, and CD86 and the chemokine receptor CCR7 [13]. DC activation by ManLAM via DC-SIGN or the mannose receptor impairs maturation and induce the production of the anti-inflammatory cytokine IL-10 and reduce IL-12 levels [9]. Di-*O*-acyl trehalose (DAT), a cell wall glycolipid of virulent mycobacteria, inhibits ConA induced T cell proliferation [14]; DAT also decreases the nitric oxide synthase (iNOS) and the production of nitric oxide by macrophages [15].

The purpose of this work was to assess the role of DAT in DC functionality. We found that DAT promotes a tolerogenic profile in DCs activated with mycobacterial antigens and TLR agonists. DAT affected DC maturation, cytokine production and upregulated Indoleamine 2,3-dioxygenase (IDO) and the expansion of regulatory T cells.

## Materials and methods

### Ethics statement

The use of animals and the experimental procedures followed in this study were approved by the Comisión Institucional para el Cuidado y Uso de Animales de Laboratorio and the Comite de Bioética of the Instituto de Investigaciones Biomédicas of the Universidad Nacional Autónoma de México. All the mice used were euthanized in CO_2_ chamber.

### Antibodies

The following monoclonal antibodies (mAb) were obtained from BioLegend (San Diego, CA, USA): MHC-I (Alexa Fluor 647, clone AF6-88.5), CD40 (PE/Cy5, clone 3/2.3), PD-L1 (PE/Cy7, clone MIH5), IDO (clone mIDO-48) and an ELISA MAX standard set for mouse IFN-γ, IL-12 (p70), IL-10, and TNF-α. From Tonbo Biosciences (San Diego, CA. USA) mAb to CD11c (PE, clone N418); MHC-II (APC, clone APC 114.15.2); CD80 (FITC, clone 16-10A1); CD86 (APC, clone GL-1); CD25 (PE/Cy7, clone PC61.5); CD4 (PE, clone GK1.5). From eBioscience (San Diego, CA, USA): anti-CD25 (Clone 61.5) and anti-CD25 biotin (clone eBio7D4).

### Purification of Di-*O*-acyl trehalose

DAT was isolated from *Mycobacterium fortuitum* ATCC 6841; the mycobacteria were cultured for 14 days in Sauton media. Non-covalently linked lipids were extracted from the filtered biomass with CHCI_3_/CH_3_OH (1:2 vol/vol) for 1 h at 50°C. The solvent recovered was kept, and the filtered biomass was treated with CHCI_3_/CH_3_OH (2:1, vol/vol) for 1 h at 50°C. Pooled glycolipid extracts were dried and suspended in CHCl_3_/CH_3_OH/H_2_O (4:2:1 vol/vol/vol). After that, the crude lipid extract was dissolved in chloroform and applied to a Florisil column (Biotecna Corp., Miami, FL, USA). The lipids eluted with CHCI_3_/CH_3_OH were monitored by thin-layer chromatography (TLC) on silica gel-60 F_254_ coated plates (E. Merck, Darmstadt, Germany) and developed with CHCI_3_/CH_3_OH/H_2_O (60:12:1, vol/vol/vol). The sugar-containing compounds were visualized by spraying the plates with 2% anthrone in concentrated H_2_SO_4_ followed by heating at 110°C. Acylated trehaloses appeared as anthrone-positive lipids (blue spots) with an Rf value of 0.37 for DAT. The lipids were purified with Sep-Pak columns (Waters, Milford, MA. USA) and analyzed by TLC to confirm DAT purification. The fractions with purified DAT were pooled, dried and subjected to the Lymulus test to verify endotoxin contamination (Lonza, Basel, Switzerland).

### Characterization of Di-*O*-acyl trehalose by Fourier transform infrared spectroscopy

The lipids were analyzed by Fourier Transform infrared spectroscopy (FTIR) and recorded in a Vector 33 FTIR spectrometer (Bruker Corporation, Billerica, MA, USA), equipped with an attenuated total reflection module. The lipid (0.5 mg) was dissolved in 200 μl CHCI_3_/CH_3_OH (9:1, vol/vol) and placed into the ATR cell FTIR spectrum for measuring in wave number range of 4000–450 cm^−1^.

### Culture of *Mycobacterium bovis*/BCG and isolation of cell walls

Mycobacteria were obtained from the American Type Culture Collection (ATCC 35733) and growth for 14 days in Sauton medium. The bacilli were treated for 1 h with CHCI_3_/CH_3_OH (1:2, vol/vol) at 50°C, to obtain delipidated cell walls. The filtered biomass was treated with CHCI_3_/CH_3_OH (2:1, vol/vol) for 1 h at 50°C. The bacilli were sonicated at 60 KHz on the ice, (20 cycles, 1 min each). To obtain lipid-free mycobacterial antigens, after sonication, the cell wall fraction was recovered by centrifugation at 20,000 RCF. A Lowry protein assay was used to quantitate protein concentration; after that, a 15% SDS-PAGE followed by immunoblot were carried out to analyze the protein profiles of delipidated and non-delipidated cell walls. PVDF membranes with transferred BCG proteins were incubated overnight at 4°C with a rabbit anti-BCG serum diluted 1:500. The membranes were incubated with a peroxidase-labeled goat anti-rabbit IgG antibody (Santa Cruz Biotechnology, Dallas, TX, USA).

### Obtention and differentiation of dendritic cells

The method used by Inaba et al. was followed [16]. The tibiae and femurs were dissected from C57BL/6 6-weeks old female mice euthanized in a CO_2_ chamber. The bone marrow was flushed with a syringe filled with RPMI 1640 medium; a lysis buffer was used to deplete red cells. Cells were growth in RPMI-1640 with 5% heat-inactivated FBS, 20 μg/mL gentamicin, 100 mL 2-mercaptoethanol (0.1 M/L), and HEPES 1M (25 ml/L). To drive DCs differentiation, 20 ng/ml recombinant GM-CSF was added to the culture medium. After three days complete culture medium was added; on day 6, immature DCs growing in conglomerates were recovered and rinsed at 453 g for 5 min with PBS. By FACS, CD11c was measured to determine DC maturation (Calibur Cytometer (Beckton Dickinson, San Diego, CA, USA).

### Assays to study the impact of Di-*O*-acyl trehalose on dendritic cell activation induced by mycobacterial antigens

For these assays, 10 μg DAT dissolved in hexane/ethanol (1:1 vol/vol) were placed in 24-well cell culture plaques; after solvent evaporation, 5 x 10^5^ DCs/ml were added to the DAT containing wells and incubated for 1 h at 37°C with 5% CO_2_. After that, 20 μg of delipidated *M*. *bovis* BCG (BCG) cell wall protein were added to the wells. After 24 h incubation at 37°C with 5% CO_2_, cells were harvested and rinsed with PBS. To analyze maturation, DCs were incubated with mAb to CD11c, MHC-I, MHC-II, CD40, CD80, and CD86. For FACS analysis, CD11c^+^ cells were gated, and a geometric mean fluorescence index was set (MIF) for maturation markers. By an ELISA method, cytokines in the culture media were measured using mAb to IL-12, TNF-α, and IL-10. Since the production of cytokines by DCs may vary with time, 6 and 24 h supernatants were analyzed.

### Effects of Di-*O*-acyl trehalose on dendritic cell maturation induced by TLR agonists

Mycobacterial antigens can activate DCs through TLRs, mainly TLR-2 and TLR-4 [17, 18]. In this study, we analyzed the impact of DAT on DCs activation induced by lipopolysaccharide (LPS) (eBioscience, San Diego, CA, USA) a TLR-4 agonist and by lipoteichoic acid (LTA) (InvivoGen, San Diego, CA, USA), a TLR-2 agonist. 10 μg DAT dissolved in hexane/ethanol (1:1 vol/vol) was added to the wells. After solvent evaporation, 5 x 10^5^ cells/ml were added to the wells and incubated for 1 h at 37°C with 5% CO_2_. After that, LPS or LTA (1 μg each) were added to the wells. After 24 h incubation at 37°C with 5% CO_2_, DCs maturation was analyzed as described above.

### Assays to quantitate Indoleamine 2,3-Dioxygenase, CD25 and PD-L1 in dendritic cells after treatment with Di-*O*-acyl trehalose, mycobacterial antigens, and TLR agonists

Recently, molecular markers of tolerogenicity have been described [6]. One of them is IDO, an immunosuppressive enzyme which expression and enzymatic activity were analyzed. DC proteins were obtained treating cells with a RIPA buffer and protease inhibitors (ThermoFischer Scientific Waltham, MA, USA); proteins were separated in 12% PAGE-SDS gels, transferred to PVDF membranes and incubated overnight with an anti-IDO mAb diluted 1:500. After that, an anti-rat IgG antibody labeled with peroxidase was added. IDO enzymatic activity was quantitated analyzing kynurenine production by a colorimetric method. Briefly, the RPMI medium was removed and 1 mL of Hank’s solution (NaCl 8 g, KCl 0.4 g, C_6_H_12_O_6_ 1 g, Na_2_HPO_4_ 0.358 g, K_2_HPO_4_ 0.6 g CaCl_2_ 0.72 g, MgSO_4_•7H_2_O 1.23 g, NaHCO_3_ 0.35 g) was added along with 10 μM L-Tryptophan (Sigma-Aldrich, St. Louis, MO. USA) and the cells were incubated at 37°C with 5% CO_2_ and supernatants were collected at 15, 30, 60 and 120 min. An equal volume of 30% trichloroacetic acid was added to supernatants and centrifuged at 8000 g for 5 minutes, 75 μl supernatant was added to an equal volume of 2% Ehrlich reagent with glacial acetic acid in a microtiter plate well; a purified L-kynurenine (Sigma-Aldrich, St. Louis, MO. USA) was used to make a standard curve. The optic density was measured at 492 nm using Asys expert plus microplate titer (Hitech GmbH, Austria). It has been reported that CD25 could have a role in tolerogenicity [19], we studied cell surface CD25 expression by FACS and soluble CD25 (CD25s) production was measured in supernatants by an ELISA method at 6 and 24 h; a recombinant CD25 (Biolegend, San Diego, CA. USA) was used to make the standard curve. Finally, the expression of the co-inhibitory molecule was analyzed by FACS.

### Induction of regulatory T lymphocytes by Di-*O*-acyl trehalose treated dendritic cells

A defining feature of tolerogenic DC is its capacity to generate regulatory T lymphocytes [6, 7]. To investigate if DAT can induce proliferation of FoxP3^+^ lymphocytes a mixed lymphocyte reaction (MLR) was carried out. Lymphocytes were obtained from spleens of C57BL/6-Tg (Foxp3-GFP)90Pkraj/J mice obtained from Jackson Laboratory (Bar Harbor, ME, USA). The spleens of these mice were passed through a cell strainer immersed in RPMI 1640 with 5% FBS. Cells were rinsed with RPMI 1640, centrifuged for 5 min at 3503 g and labeled with an anti-CD4 PE-labeled mAb diluted 1:2000 in PBS/FBS for 30 min. After extensive rinsing CD4^+^FoxP3^+^ and CD4^+^FoxP3^-^ populations were acquired by cell sorting using a FACSAria cytometer (BD Pharmigen, Franklin NJ, USA). Fluorescence dilution method was used to study T lymphocytes proliferation; purified lymphocytes were stained with 5 mM red fluorescent Dye eFluor 670 (Invitrogen Molecular Probes, Eugene OR, USA). Stained lymphocytes were cocultured with treated DCs in a 10:1 proportion in U-bottom 96-well plates. After three days, cells were harvested and marked with a mAb anti-CD25 labeled with PE/Cy7. We analyzed cell proliferation of CD4^+^CD25^+^FoxP3^+^ and CD4^+^CD25^-^FoxP3^-^ populations using FlowJo software; a division percentage was set. Also, IL-10 and IFN-γ were analyzed by an ELISA method in 72 h supernatants.

### Statistical analysis

The GraphPad PRISM software (version 5.01; San Diego, CA, USA) was used for statistical analysis. Data are expressed as mean ± standard error. Kolmogorov-Smirnoff normality tests were performed, and the results were analyzed with an impaired t-Student’s test and Mann Whitney test.

## Results

### Isolation of Di-*O*-acyl trehalose and mycobacterial cell walls

**Fig 1:**
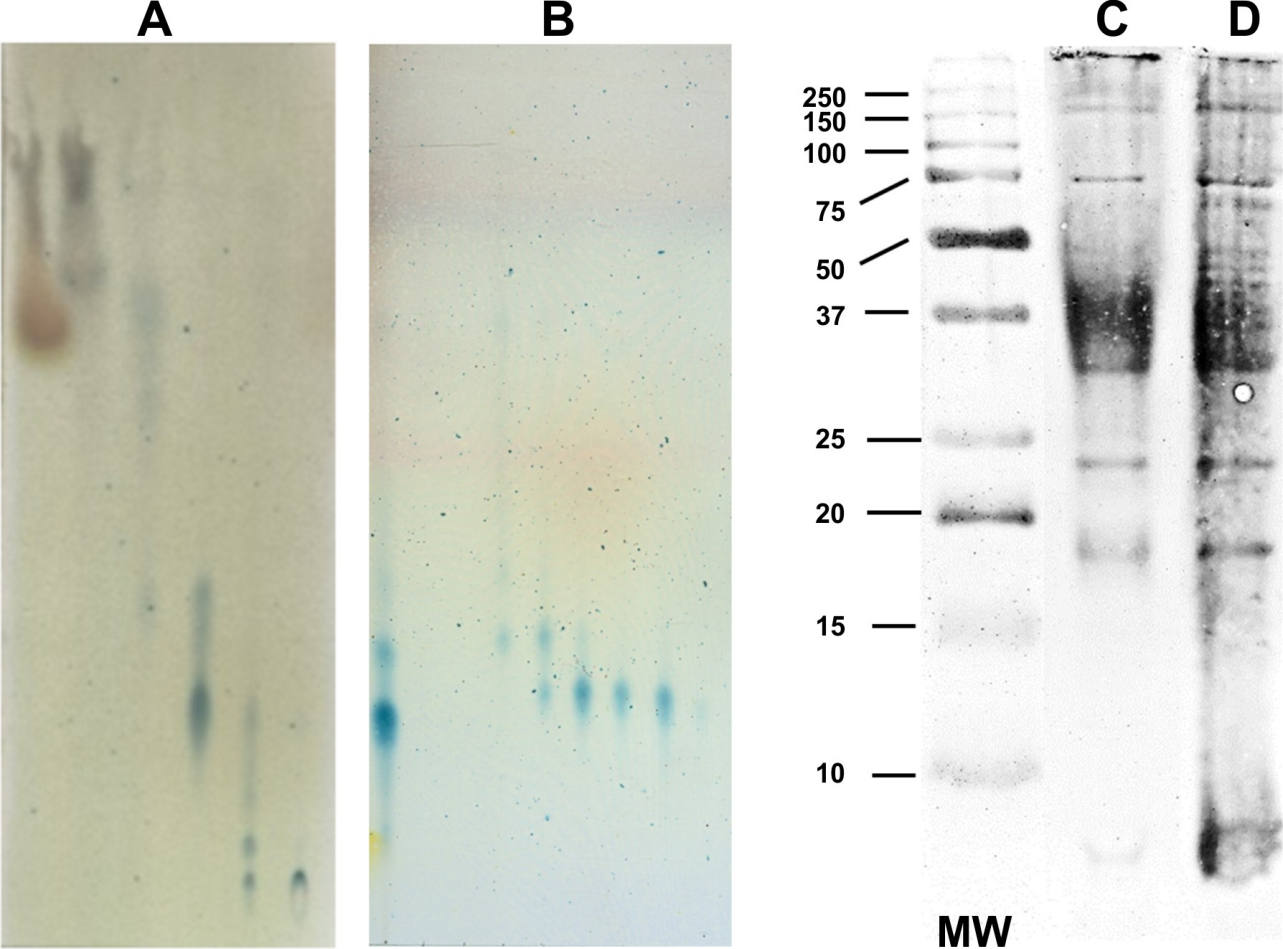
Isolation of Di-*O*-acyl trehalose and mycobacterial antigens. Thin-layer chromatography of *M*. *fortuitum* ATCC 6841 unfractionated lipids extracted with CHCl_3_/CH_3_OH/H_2_O (A). The spots with Rf value of 0.37 correspond to isolated DAT (B). Immunoblot shows protein profiles of untreated BCG cell-walls (C) and cell walls delipidated with CHCl_3_/CH_3_OH/H_2_O (D).

Trehaloses are glycolipids of virulent mycobacteria that may behave as virulence factors [14, 15]. DAT was purified from *M*. *fortuitum*, saprophytic mycobacteria that produce DAT similar to that of Mtb [14]. Total lipids were isolated from the biomass by Folch washing with CHCl_3_/CH_3_OH/H_2_O. Elution of the extract was carried in a Fluorisil column and monitored with TLC (Fig 1A). Eluted fractions containing blue spots and an Rf value of 0.37 were pooled and eluted in a Sep-pak column to purify DAT (Fig 1B). DAT was analyzed by Fourier’s transformed infrared spectroscopy showing the corresponding infrared spectrum as reported previously [15]. Endotoxin was not detected by the Limulus amebocyte lysate test. Mycobacterial antigens for DC activation assays were obtained from BCG cell walls that were delipidated with chloroform/methanol to eliminate cell surface glycolipids that could alter DC activation. Immunoblot was performed to verify the protein content of the walls with a rabbit antiserum to BCG. In non-delipidated cell walls, it was observed a diffuse, ill-defined band suggestive of lipoarabinomannan (Fig 1C). In delipidated cell walls protein bands were incremented in number and intensity (Fig 1D).

### Di-*O*-acyl trehalose interferes with the maturation of dendritic cells activated with mycobacterial antigens

**Fig 2:**
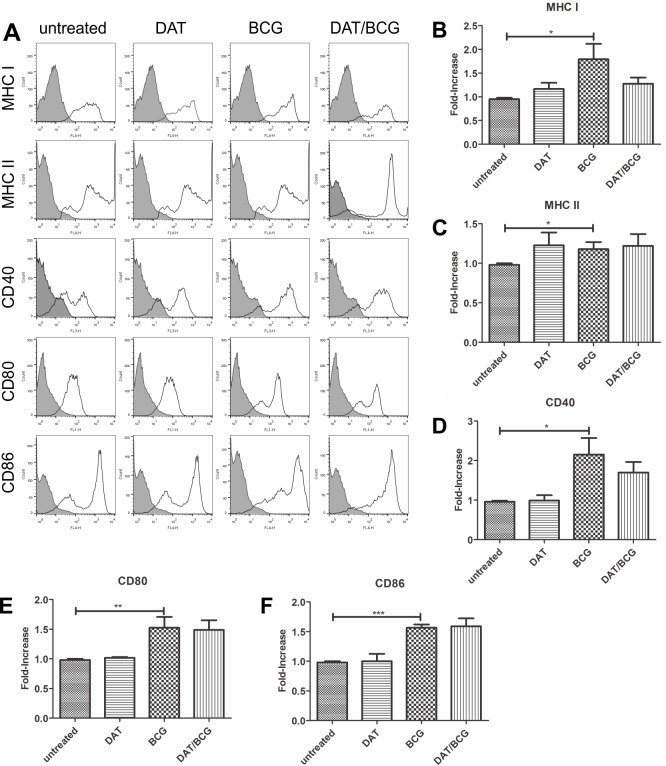
Di-*O*-acyl trehalose interferes with the maturation of DCs activated with BCG antigens. DC bone marrow precursors cultured with GM-CSF for six days were incubated with 20 μg *M*.*bovis*/BCG cell wall antigens for 24 h; the maturation markers were analyzed by FACS in CD11c^+^ cells (A). Increased expression of MHC-I, MHC-II and costimulatory molecules CD40, CD80 and CD86 are observed (p < 0.05; unpaired t Student’s test) (B-F). To determine the effects of DAT on DC maturation induced by BCG antigens, DC were treated with 10 μg DAT for 1 h and then with mycobacterial antigens. DAT diminished the increased expression of MHC-I and CD40 induced by BCG (not significant) (B, D).

Immature DCs were obtained from bone marrow precursors of C57BL/6 mice. After six days of culture with GM-CSF, DC formed loosely organized no- adherent cell aggregates and showed a distinctive morphology with cytoplasmic protrusions resembling dendrites (not shown). At six days CD11c expression varied from 70 to 80% and MHC-II from 60 to 70%. For DC activation cell walls were obtained from *M*. *bovis* /BCG bacilli that had been delipidated with CHCl_3_/CH_3_OH/H_2_O to get rid of glycolipids that could inhibit DC maturation. BCG cell walls contain antigenic proteins that efficiently trigger DC maturation [17]. Analysis of antigen presentation and costimulatory molecules was carried out on CD11c+ cells, and MIF was analyzed (Fig 2A). In DCs incubated with BCG cell walls for 24 h, there was a marginal increase in the expression of antigen presentation molecule MHC-I and costimulatory molecules CD40, CD80 and CD86 (Fig 2B, 2D, 2E and 2F). Pretreatment of cells with 10 μg DAT inhibited partially the upregulation of MHC-I and CD40 induced by BCG (Fig 2B and 2D).

### Dendritic cells activated with mycobacterial antigens and Di-*O*-acyl trehalose modulate cytokine production

**Fig 3:**
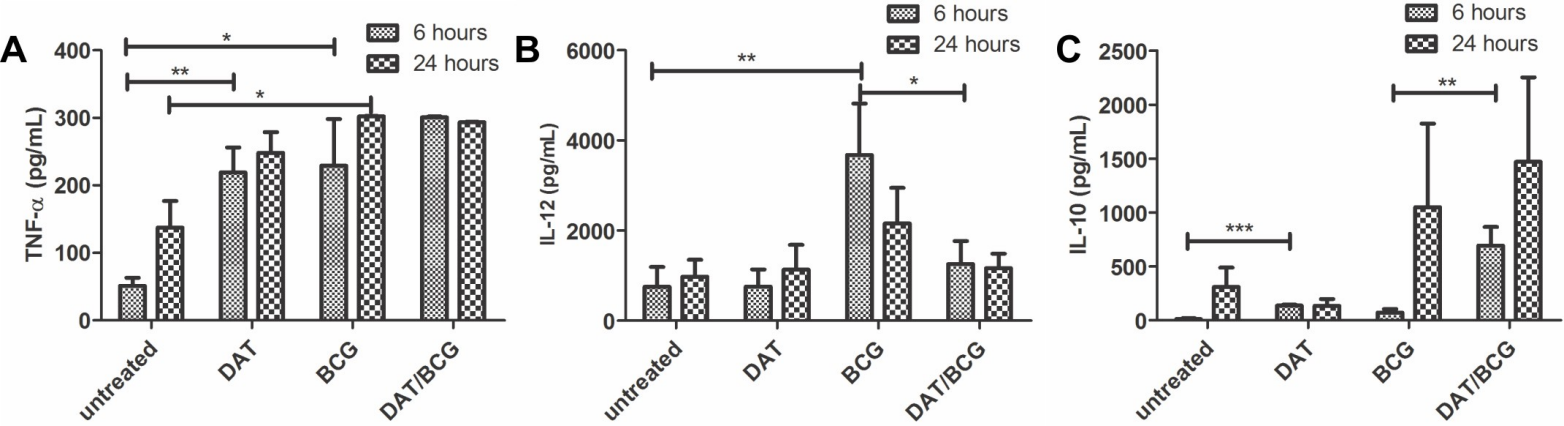
Di-*O*-acyl trehalose increases IL-10 production in dendritic cells activated with mycobacterial antigens. The production of cytokines was analyzed by ELISA in 24 h supernatants. BCG upregulates TNF-α and IL-12 (p < 0.05; unpaired t Student’s test) (A, B); the production of IL-10 was not altered. DC pretreated with DAT and then with BCG increase IL-10 production (p < 0.0001; unpaired t Student’s test) (C). DAT alone upregulated TNF-α (p < 0.05 unpaired t Student’s test) (A). The results shown were obtained in five independent experiments.

The autocrine production of proinflammatory cytokines by DCs is of prime importance in the immune response to mycobacteria [2]. Supernatants of 6 and 24 h of culture were analyzed by ELISA. In response to BCG, DCs upregulated the production of proinflammatory cytokines IL-12 and TNF-α (Fig 3A and 3B). An interesting finding was the upregulation of TNF-α by DCs treated with DAT alone. It was also of interest that pretreatment of cells with DAT markedly reduced the increased production of Il-12 induced by BCG. IL-10, a prototypic anti-inflammatory cytokine, plays a crucial role in tolerogenicity [6, 7]. BCG antigens upregulated IL-10 (Fig 3C). DAT alone augmented IL-10 at 6 h of culture, and when it was given in addition to BCG antigens, a synergistic effect in IL-10 production was observed.

### Di-*O*-acyl trehalose down-modulates the maturation of dendritic cells induced by TLR agonists

**Fig 4:**
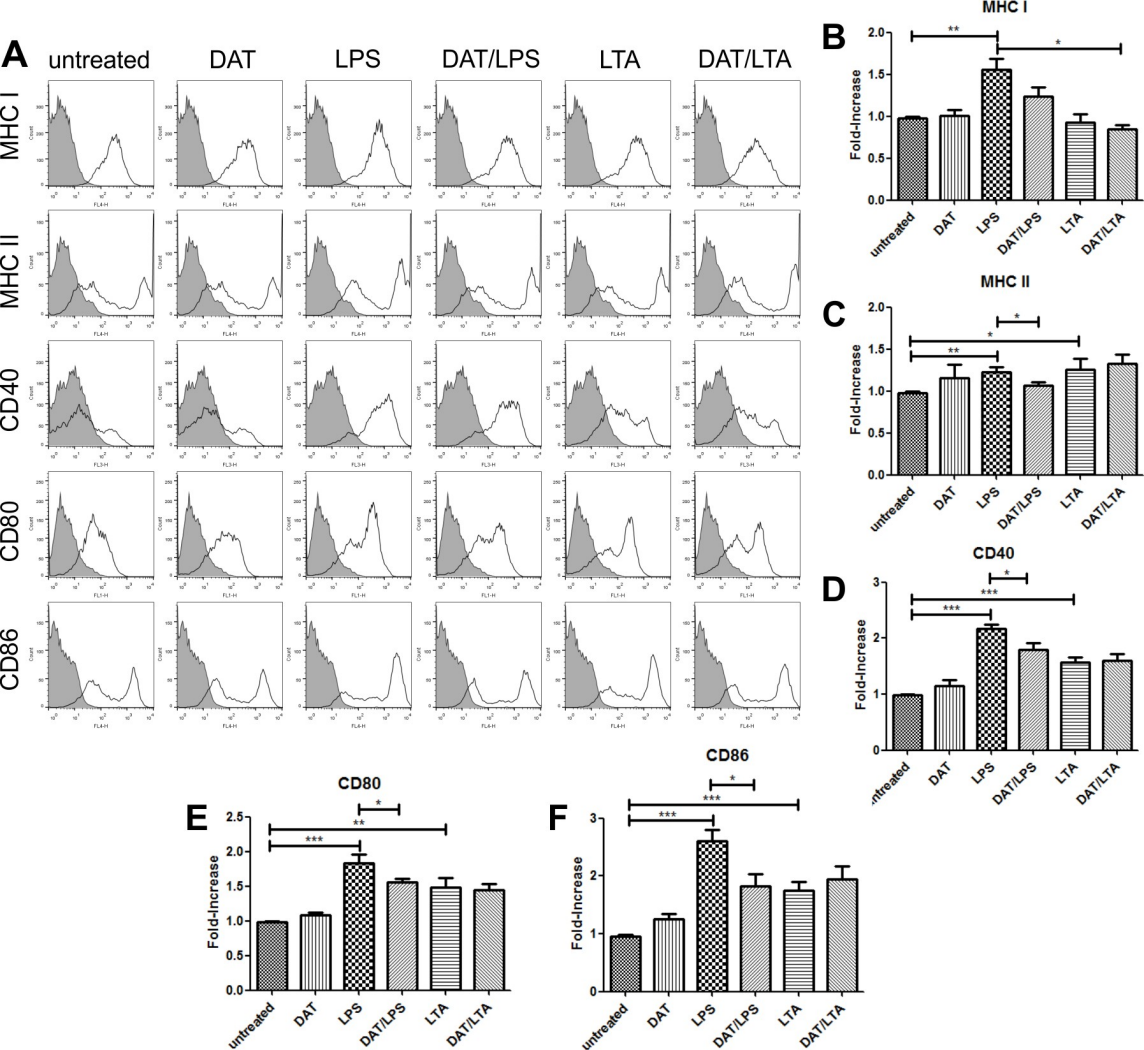
Di-*O*-acyl trehalose antagonizes the effects of LPS on DCs maturation. LPS, a TLR-4 agonist increase the levels of MHC-I, MHC-II, CD40, CD80 and CD86 (p < 0.005; unpaired t Student’s test) (B-F). Similar effects are obtained with LTA, a TLR-2 agonist. Pretreatment of with 10 μg DAT for 1 h diminished the effects of LPS on DC maturation significantly (p < 0.05; unpaired t Student’s test) (B-F). DAT had no effects on LTA induced DC maturation.

DCs sense microbial products through a variety of cell surface receptors among which TLRs have been the best studied [6, 8]. Mtb has components that behave as TLR agonists particularly for TLR-2 and TLR-4 [20, 21, 22], and there are mycobacterial glycolipids that modulate the effects of these agonists on DCs [20, 22]. Hence, we considered of interest to study the impact of DAT on DCs activated by LPS a TLR-4 agonist and LTA an agonist for TLR-2. By flow cytometry, an analysis of the expression of MHC-I, MHC-II, CD40, CD80 and CD86 was carried out (Fig 4A). We found that LPS and LTA efficiently induced DCs maturation upregulating antigen presentation molecules MHC-I and MHC-II and the costimulatory molecules CD40, CD80 and CD86; interestingly the maturation induced only by LPS was diminished when cells were pre-treated with DAT (Fig 4B–4F).

### Di-*O*-acyl trehalose downregulates IL-12 production and increases the release of IL-10 by dendritic cells induced to maturation with lipopolysaccharide

**Fig 5:**
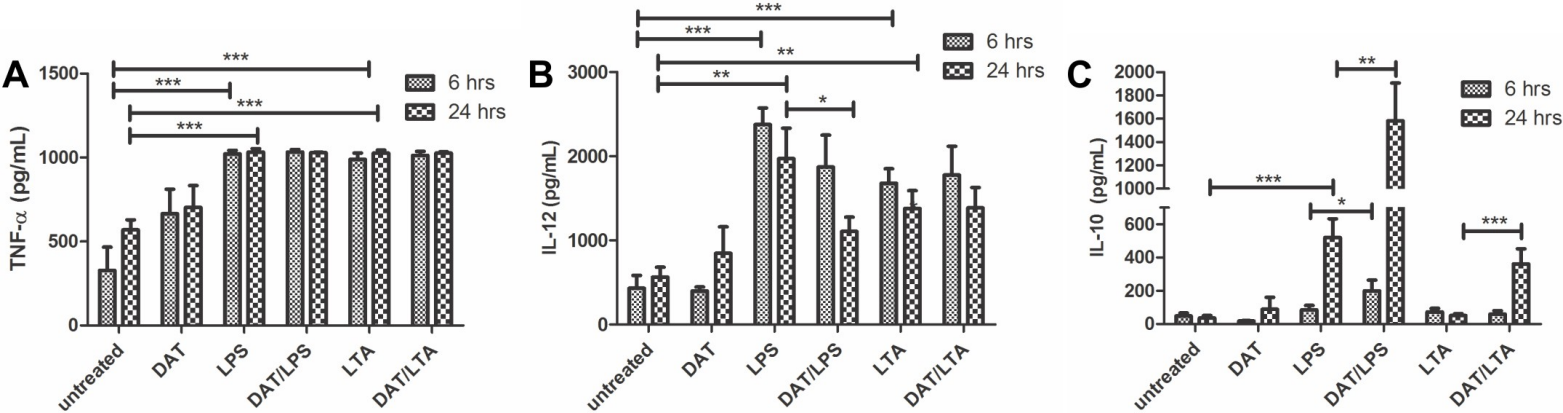
Di-*O*-acyl trehalose counteracts the effects of LPS and LTA cytokine production by dendritic cells. Regarding cytokines, LPS treated DCs upregulated TNF-α, IL-12, and IL-10 (p ≤ 0.0005; unpaired t Student’s test) (A-C). LTA upregulated TNF-α and IL-12 (p < 0.001; unpaired t Student’s test) (A-C). DAT modified cytokine release triggered by LPS reducing greatly IL-12, and increasing IL-10 (p < 0.005; unpaired t Student’s test) (B, C). The results shown were obtained in five independent experiments.

An ELISA method was carried out to study cytokine production at 6 and 24 h. LPS upregulated the production of TNF-α, IL-12, and IL-10 at 6 and 24 h significantly (Fig 5A–5C). LTA upregulated TNF-α and IL-12 but did not modify IL-10 production. The impact of DAT on cytokine production induced by LPS was of note. DCs treated with DAT and then with LPS markedly reduced IL-12 production at 24 h while a significant increment in IL-10 production was observed.

### Di-*O*-acyl trehalose induces Indoleamine 2,3-Dioxygenase in immature DCs and DCs activated with LPS and LTA

**Fig 6:**
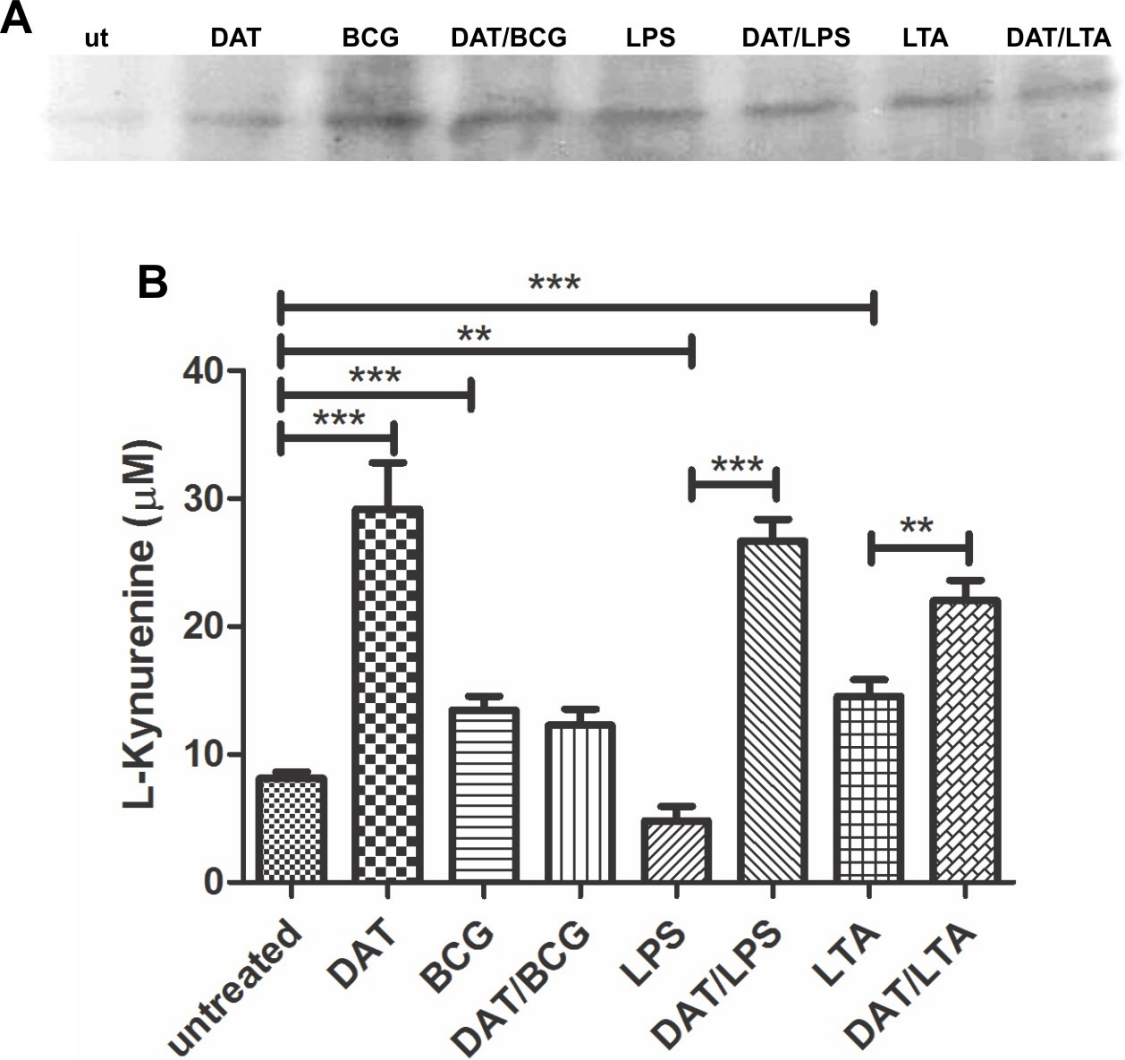
Di-*O*-acyl trehalose upregulates the expression of Indoleamine, 2,3 Dioxygenase in DCs activated with mycobacterial antigens and TLR agonists. IDO expression and enzymatic activity were analyzed by immunoblot and by measuring kynurenine production respectively. Immunoblot of DC proteins extracted with a RIPA buffer and separated in 12% PAGE-SDS gels demonstrates the expression of IDO under all experimental conditions, being in DCs activated with BCG most evident (A). In 2 h analysis DAT alone induced the highest levels (p = 0.0004; unpaired t Student’s test) followed by BCG and LTA (p = 0.0004 and p = 0.0003, respectively; unpaired t Student’s test). LPS failed to upregulate kynurenine, but in combination with DAT, a highly significant increase was demonstrated (p < 0.0001; unpaired t Student’s test); also, DAT potentiated LTA effects (p = 0.0121; unpaired t Student’s test) (B). The results shown were obtained in seven independent experiments.

In addition to low levels of antigen presentation, costimulatory molecules, and upregulation of anti-inflammatory cytokines, tolerogenic DCs can present molecules that have been considered markers of tolerogenicity. Among these is IDO, an immune-regulatory enzyme that participates in immunological tolerance [23]. By immunoblot, IDO expression was not detected in immature DCs, but in cells activated with mycobacterial antigens and TLR agonists, a reactive band was detected that was more intense in DCs induced to maturation with BCG walls (Fig 6A). An analysis of kynurenine production after 24 h DC activation with BCG and TLR agonists showed that DAT alone and to lesser extent BCG and LTA upregulated IDO. LPS alone did not upregulate kynurenine however when DCs were pretreated with DAT, and then with LPS, a significant increment was observed. A similar synergistic effect was observed in cells co-treated with DAT and LTA (Fig 6B).

### Analysis of coinhibitory molecules induced by Di-*O*-acyl trehalose

**Fig 7:**
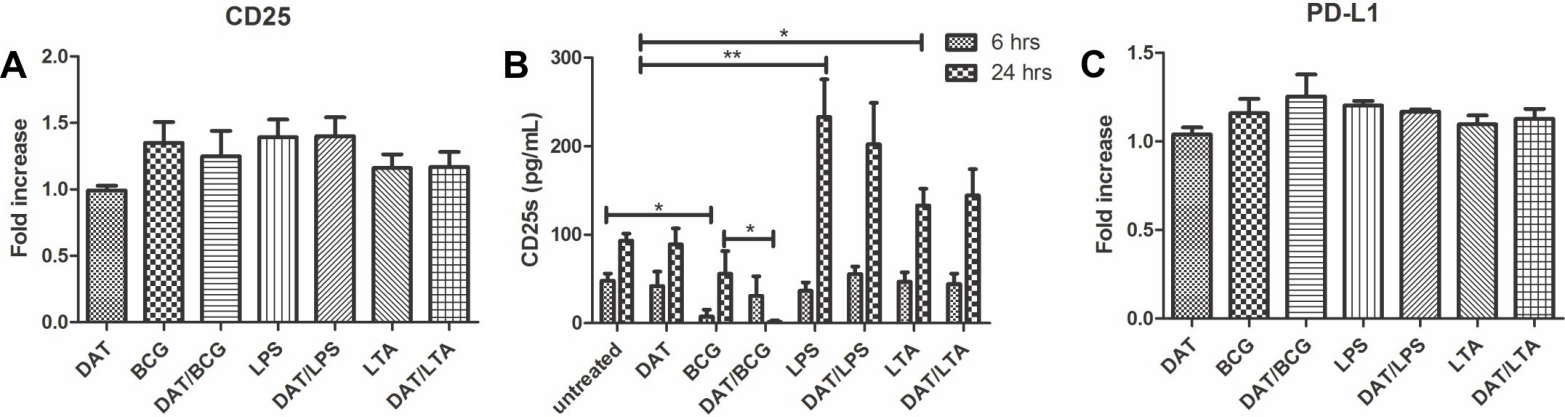
Effects of Di-*O*-acyl trehalose in the expression of coinhibitory molecules. Since an immunoregulatory function for CD25 has been proposed, we analyzed its expression by FACS and ELISA. A small increase in CD25 surface expression in DCs treated with BCG and TLR agonists with or without DAT (A). ELISA of supernatants showed increased levels of CD25s in DCs treated with LPS and with LTA at 24 h of culture (p < 0.05; unpaired t Student’s test) (B). The expression of PD-L1 was similar in basal and experimental conditions (C).

CD25, the alpha subunit of the IL-2 receptor, has been considered a marker of tolerogenic DC and recently its coexpression with IDO has been reported [19]. FACS revealed a nonsignificant upregulation of cell-surface CD25 in CDs treated with mycobacterial antigens and TLR agonists, that was not affected by DAT pretreatment (Fig 7A). ELISA of 24 h culture supernatants showed augmented levels of CD25s by CD treated with LPS and LTA, which were not modified by pretreatment with DAT (Fig 7B). Finally, FACS showed that surface PD-L1, the ligand that interacts with PD-1 in T cells during the immunological synapse [6], was marginally increased by DCs treated BCG, LPS and by DAT/BCG (Fig 7C).

### Dendritic cells treated with Di-*O*-acyl trehalose promote the expansion of regulatory T lymphocytes

**Fig 8:**
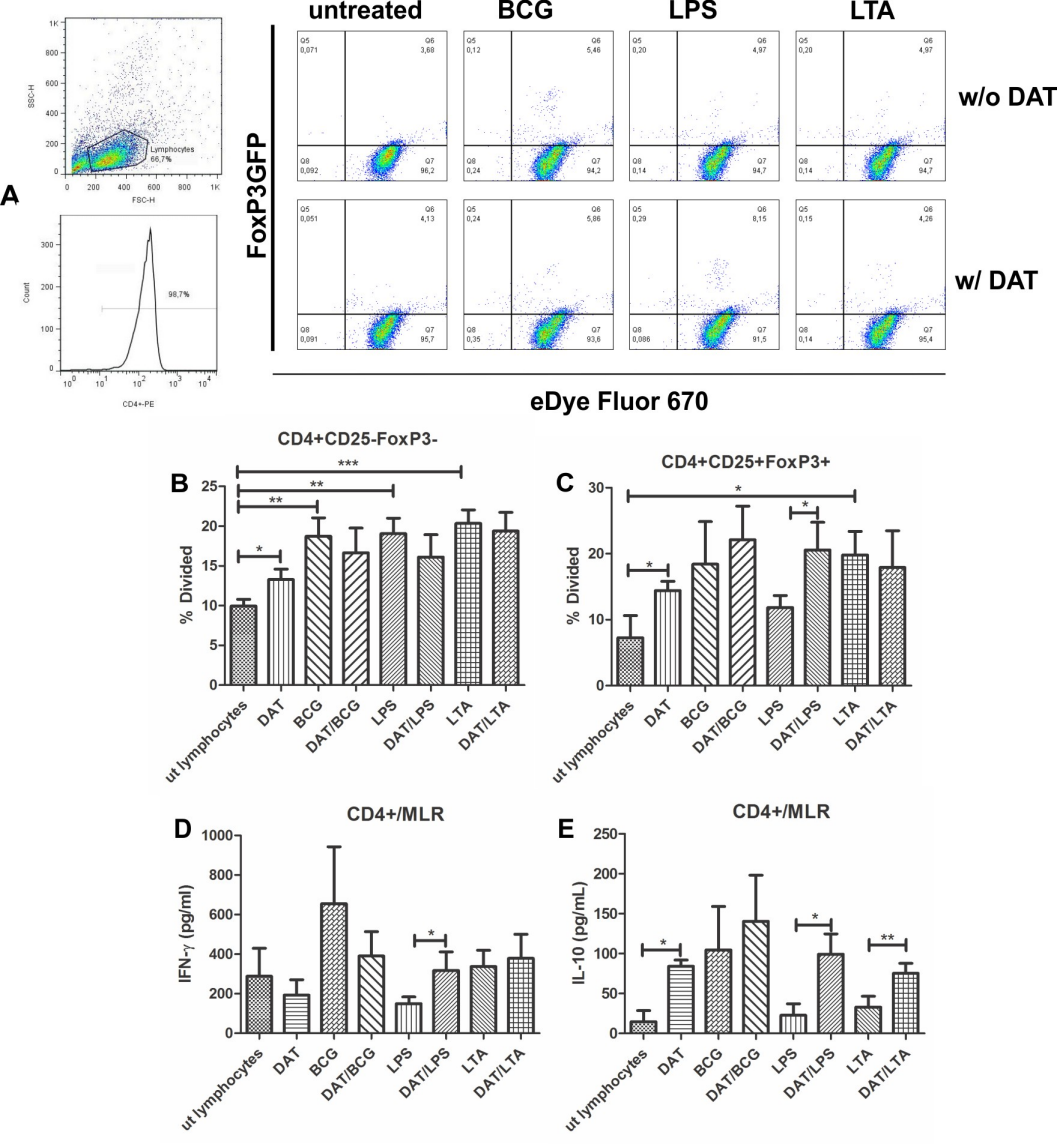
Di-*O*-acyl trehalose promotes the expansion of regulatory T lymphocytes. We studied the capacity of DCs activated with *M*. *bovis*/BCG antigens and TLR agonists with or without preincubation with DAT. DCs were cocultured with lymphocytes CD4^+^CD25^-^FoxP3^-^ and CD4^+^CD25^+^FoxP3^+^ lymphocytes obtained by cell sorting from Foxp3GFP^+^ transgenic mice. The proliferation was measured by a dilution method with eDye Fluor 670 (A). Proliferation of FoxP3^-^ lymphocytes was observed in cocultures with DCs activated with DAT, BCG antigens, and LPS (p < 0.05; unpaired t Student’s test) (B). Proliferation of regulatory FoxP3^+^ lymphocytes was induced by CDs activated with LTA (p = 0.0169; unpaired t Student’s test); BCG and LPS activated DCs increase the proliferation of regulatory T lymphocytes, but results do not reach statistical significance. Of interest, DAT alone triggered the expansion of regulatory lymphocytes (p = 0.0463; unpaired t Student’s test), and when given together with LPS a positive synergistic effect was observed (p = 0.476; Mann Whitney test) (C). The production of cytokines in a mixed lymphocyte reaction with purified CD4^+^ was quantitated by ELISA in cocultures of DCs with splenic lymphocytes from Foxp3GFP^+^ transgenic mice. Increased release of IFN-γ is observed with DCs activated with BCG antigens (D). The production of the anti-inflammatory cytokine IL-10 was upregulated in cocultures of lymphocytes with DCs treated with BCG, DAT/BCG, DAT/LPS and DAT/LTA but results were not statistically significant. When DCs were pretreated with DAT alone or DAT and LPS or LTA a synergistic effect in IL-10 production was observed (p < 0.05; Mann Whitney test) (E). The results of five independent experiments are shown.

The ability to expand regulatory T lymphocytes is a distinctive feature of tolerogenic DCs [6, 7]. Since the above findings seem to indicate a tolerogenic profile for CDs treated with DAT, we wanted to know if these cells triggered the proliferation of CD4^+^C25^+^FoxP3^+^ T lymphocytes. For this purpose, Foxp3GFP^+^ transgenic mice were obtained and the spleen CD4^+^CD25^-^FoxP3^-^ and CD4^+^CD25^+^FoxP3^+^ T cell populations were purified by cell sorting. The isolated lymphocytes were incubated with DCs that were exposed to DAT and then activated with TLR agonists or mycobacterial antigens as described before. Cell proliferation was measured by a dye dilution procedure with eDye Fluor 670 and analyzed with a FlowJo program (Fig 8A). With CD4^+^CD25^-^FoxP3^-^ lymphocytes, there was a significant proliferative response with DCs treated with DAT alone, BCG antigens, DAT/BCG and LPS (Fig 8B). With CD4^+^CD25^+^FoxP3^+^ lymphocytes, there was a significant increment in cell proliferation when lymphocytes were incubated with DCs activated with DAT and LTA (Fig 8C). Increased proliferation of regulatory lymphocytes was also observed with DCs activated with BCG, DAT/BCG, and DAT/LPS. It was interesting the synergistic effect of DAT upregulating the proliferation of regulatory T cells induced by LPS activated DCs and the triggering of FoxP3^+^ T cells by DCs treated with DAT alone. IFN-γ and IL-10 were measured by ELISA in DC/spleen lymphocytes coculture supernatants. Increased levels of IFN-γ were observed only with DCs activated with BCG (Fig 8D). The production of the anti-inflammatory cytokine IL-10 was upregulated by lymphocytes cocultured with DCs treated with DAT, BCG, and DAT/BCG. When these DCs were pretreated with DAT, and then with LPS or LTA a positive synergistic effect in IL-10 production was observed (Fig 8E).

## Discussion

DAT is glycolipid present in the cell wall of saprophytic and virulent mycobacteria that possess the ability to modulate T cell immunity and MOs functions [15, 23]. Although whether it is used by Mtb to counteract the function of DC has not been investigated. In this study, we found that DAT induces a tolerogenic phenotype in bone marrow-derived DCs exposed to mycobacterial antigens and TLR agonists. These findings are relevant since the interaction of DCs with Mtb is of prime importance in the development of protecting T cell immunity in TB [24, 25, 26]. Together with other cells DC infiltrate in great numbers tuberculous granulomas [5]; DC with engulfed bacilli may migrate to draining lymph nodes to process ingested microbes generating immunogenic peptides that are presented to naïve T cells [1, 2]. It has been shown DC exposed to mycobacteria or their isolated components can be activated to orchestrate antimycobacterial immunity [3, 24, 25]. However, the role of DCs in the control of the TB is questioned by observations showing that mycobacteria and some cell wall glycolipids can impair their maturation [4, 5].

In this study, we found that in DCs activated with mycobacterial antigens, DAT had no significant effects on antigen presentation and costimulatory molecules, although it altered markedly autocrine cytokine production. The high production of the proinflammatory cytokine IL-12 by DCs activated with BCG cell walls diminished to almost basal values by pre-treatment with DAT. In contrast, production of the anti-inflammatory cytokine IL-10 increased in CDs treated with DAT alone or together with mycobacterial antigens. The maturation of DC is a highly complex process that can occur when cell surface receptors interact with mycobacterial antigens. In this process participate receptors of the Toll family, especially TLR-2 and TLR-4, for which some mycobacterial agonists have been identified [18, 26].

We studied the effects of DAT on DC activation induced by LPS and LTA. The best characterized TLR-4 agonist is LPS, a glycolipid that activates DCs with high efficiency [21]. In this study, activation with LPS induced DC maturation with high levels of MHC-I, MHC-II, and costimulatory molecules CD40, CD80, and CD83; pre-treatment of cells with DAT diminished these effects. These observations could be relevant since MHC I and MHC II are key maturation markers involved in the activation of the acquired T cell immunity that is of critical importance in TB. Through MHC-II antigenic peptides derived from phagocytosed mycobacteria are presented by DCs to CD4 T cells to generate a protective TH1, TH17 and TH23 response [2, 24]. Downregulation of MHC-I by DAT could also have an adverse effect on antimycobacterial immunity; it has been shown that phagocytosed mycobacteria may reach the cytosol and that CD8 T cells are activated via cross-presentation through MHC-I [27].

The costimulatory molecules play a central role in the antigen presentation process and autocrine cytokine production. CD40 interacts with CD40L enhancing MHC-II and the production of pro-inflammatory cytokines [28, 29, 30]. Studies are showing that CD40 and Th1 and Th17 responses can be impaired by mycobacteria [30]. Down-regulation of costimulatory molecules, CD80 and CD86, could also have a negative effect on antigen presentation given the role these molecules play in the immunologic synapse interacting with CD28 [3].

As observed with mycobacterial antigens, DAT altered the autocrine production of cytokines in DC activated with TLR agonists. LPS promoted the production of proinflammatory cytokines TNF-α and Il-12 efficiently, an effect that was reverted when DCs were pre-treated with DAT. In contrast, IL-10 was upregulated in cells treated with DAT and LPS conjointly. As far as LTA, this TLR-2 agonist incremented costimulatory molecules and modestly TNF-α and IL-12; given together with DAT the production of TNF-α and IL-12 was potentiated but no that of IL-10. The downregulation of IL-12 in DC activated with mycobacterial antigens and LPS is of interest; IL-12, a proinflammatory cytokine produced by DC after phagocytosis of mycobacteria plays a role in the development of acquired antimycobacterial immunity. This view is supported by studies with IL-12 knockout mice which were highly susceptible to *M*. *tuberculosis* infection [30]. The high production of IL-10 by DCs exposed to DAT was another interesting finding. IL-10 is an anti-inflammatory that promotes tolerogenic DC, impairs the production of pro-inflammatory cytokines and induces the activation and expansion of regulatory T cells [31].

The above findings suggest the promotion of tolerogenic DC by DAT which was strengthened by the demonstration of IDO activity in DCs treated with DAT. High levels of L-kynurenine were induced with DAT alone or in conjunction with mycobacterial antigens and TLR agonists. IDO catalyzes the oxidation of L-tryptophan to L-kynurenine, thus depriving T cells of tryptophan an essential amino acid critical for T cell activation [32]. Recently, this immunosuppressive enzyme has focused much interest for the role it plays in DC tolerogenicity in mycobacterial infections. It has been demonstrated that mycobacteria upregulate IDO in macrophages and DCs *in vitro* and lung granulomas of infected mice and macaques [33, 34]. IDO is associated with poor antimycobacterial T cell responses and with the expansion of regulatory T cells that favors persistence of the infection [33].

A key attribute of tolerogenic DC is the ability to trigger the differentiation and expansion of regulatory T cells with the CD4^+^CD25^+^FoxP3^+^ phenotype which is critical in peripheral tolerance [6, 7]. Herein, we found that DAT alone and in conjunction with LPS were involved in the expansion of regulatory T cells. Several factors participate in the generation of these cells. One of great interest is the upregulation of IDO by DC and the associated production of kynurenine a tryptophan metabolite that induces the transcription of the aryl hydrocarbon receptor (AhR) which triggers the proliferation of FoxP3^+^ regulatory T cells [32]. Another factor is IL-10 that stimulate the generation of regulatory T cells and induce immature DCs to become tolerogenic [31].

Our current observations confirm the role of DAT as a virulence factor; this glycolipid belongs to a family of trehalose containing glycolipids which recently have acquired relevance for their role in infection [14, 15]. The acyl trehaloses of *M*. *tuberculosis* include sulfatides, trehalose dimycolates, tri-acyl trehaloses, and di-acyl trehaloses [35]. These glycolipids are located in the outer membrane of the mycobacterial cell envelope, a strategic location to interact with host cells. Only recently the role of acylated trehaloses as virulence factors has been unraveled. DAT downregulates T-cell proliferation and the production of Th-1 cytokines; this effect is associated with disruption of the MAPK signaling pathway [14, 36]. DAT also affects macrophage functions, downregulating iNOS and nitric oxide production [15].

In conclusion, in this study, we have shown that DAT a glycolipid present in the cell wall of virulent mycobacteria induces a tolerogenic phenotype in bone marrow-derived DCs. Our current observations are in keeping with studies showing that the cell wall of virulent mycobacteria is endowed with a variety of glycolipids that behave as virulence factors; some of these glycolipids can interfere with a central immune function that is the activation of the adaptative response by DC, the master antigen presenting cell.
